# Formation of smectic phases in binary liquid crystal mixtures with a huge length ratio

**DOI:** 10.3762/bjoc.8.124

**Published:** 2012-07-19

**Authors:** Nadia Kapernaum, Friederike Knecht, C Scott Hartley, Jeffrey C Roberts, Robert P Lemieux, Frank Giesselmann

**Affiliations:** 1 Institut für Physikalische Chemie, Universität Stuttgart, D-70569 Stuttgart, Germany; 2 Department of Chemistry, Queen’s University, Kingston, Ontario, Canada

**Keywords:** bidispersity, liquid crystals, phase diagrams, smectic phases, smectic F phase, structure and dynamics

## Abstract

A system of two liquid-crystalline phenylpyrimidines differing strongly in molecular length was studied. The phase diagram of these two chemically similar mesogens, with a length ratio of 2, was investigated, and detailed X-ray diffraction and electrooptical measurements were performed. The phase diagram revealed a destabilization of the nematic phase, which is present in the pure short compound, while the smectic state was stabilized. The short compound forms smectic A and smectic C phases, whereas the longer compound forms a broad smectic C phase and a narrow higher-ordered smectic phase. Nevertheless, in the mixtures, the smectic C phase is destabilized and disappears rapidly, whereas smectic A is the only stable phase observed over a broad concentration range. In addition, the smectic translational order parameters as well as the tilt angles of the mixtures are reduced. The higher-ordered smectic phase of the longer mesogen was identified as a smectic F phase.

## Introduction

**Figure 1:**
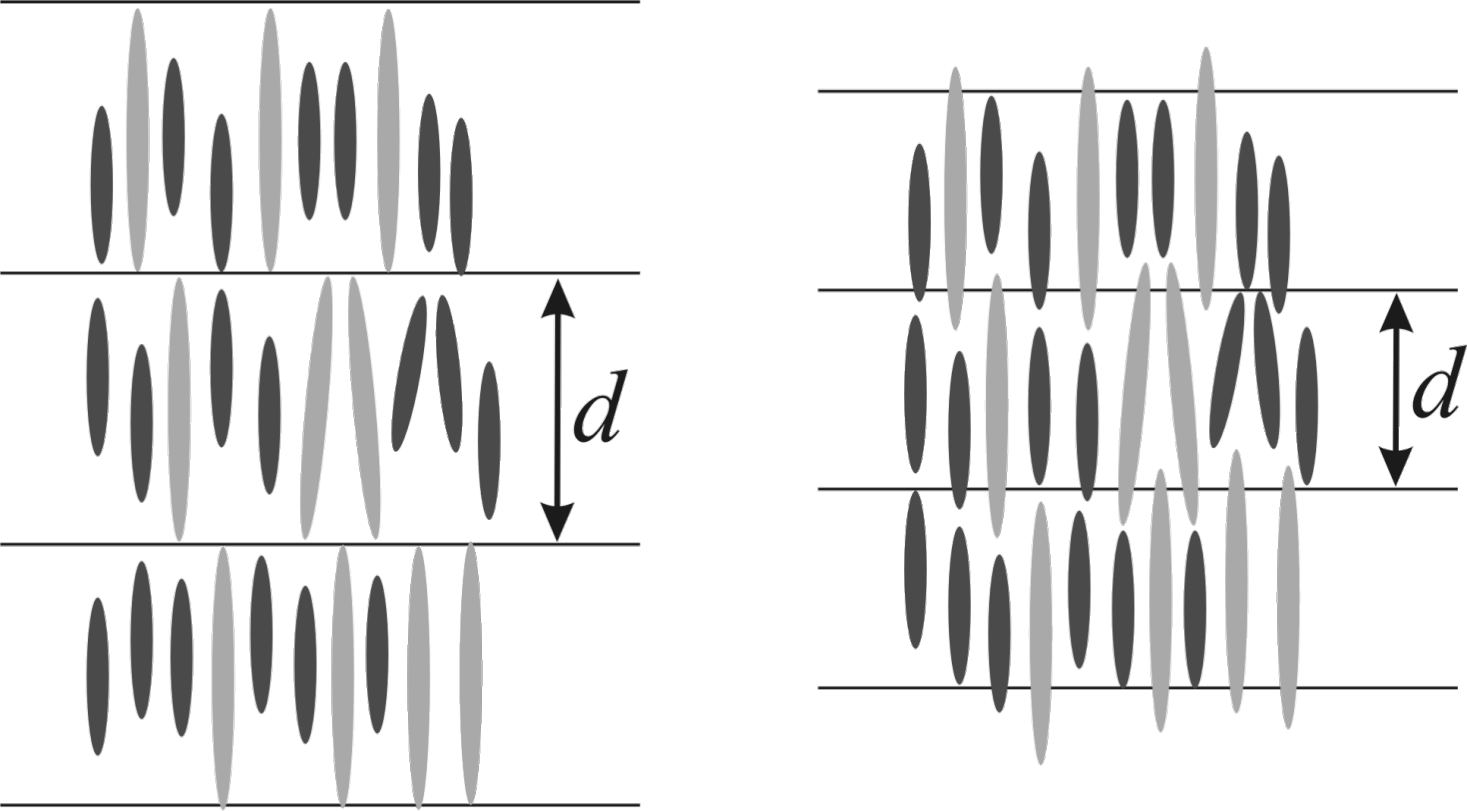
Smectic A phase of a mixture of two mesogens differing strongly in molecular length. Left: The layer spacing corresponds to the length of the long mesogen; no out-of-layer fluctuations occur. This leads to a lot of unfavorable free volume. Right: By reducing the smectic translational order the molecules are now densely packed. This leads to extensive out-of-layer fluctuations. Reproduced after [1].

The mixing of different liquid crystals is a common technique to tailor their properties for specific applications. In particular, the mixing of two mesogens that are quite different from each other can strongly change the properties and the phase behavior of the mixtures compared to the pure compounds. We recently reported a systematic study of mixtures of mesogens differing in molecular length [1–2]. In these studies, chemically similar mesogens with length ratios ranging from 1 to 1.8 were investigated. In the systems with a large difference in length, strong changes in the phase diagram were observed. The nematic phase was destabilized while the smectic state was stabilized. The temperature range of the SmC phase, which was the dominant phase in most of the pure compounds, was reduced while the SmA phase became broader. In the systems with a length ratio of 1.8, the smectic A phase was the only stable mesophase over a broad temperature and concentration range. Furthermore, a decrease of the tilt angle θ of the SmC phase, as well as a reduced smectic order parameter Σ in the SmA phase, were observed for these systems. We showed that the ordering of the bidisperse molecules in a SmA phase can be explained by extensive out-of-layer fluctuations (Figure 1) [1–2]. In a SmA phase of strongly bidisperse molecules, energetically unfavorable free volume remains between the layers in the absence of these fluctuations; this free volume is minimized in the “out-of-layer fluctuations” model by thermal translational fluctuations of the molecules along the layer normal. This model fits very well with the experimental data and reflects the molecular ordering in the smectic phases of bidisperse molecules best.

**Scheme 1:**
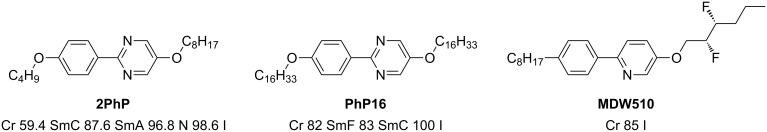
Chemical structures and phase-transition temperatures of the mesogens **2PhP** and **PhP16** and of the chiral dopant **MDW510**.

**Figure 2:**
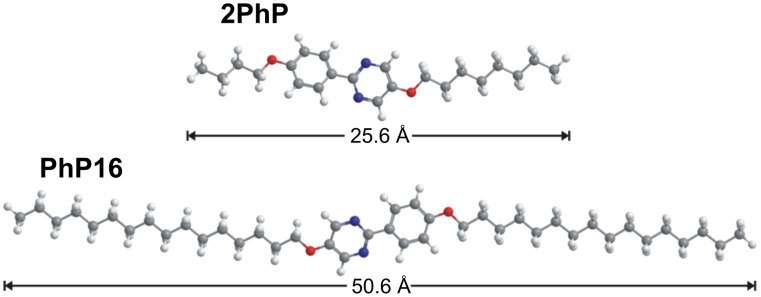
Molecular structures and molecular lengths of **2PhP** and **PhP16.** The longer mesogen **PhP16** is approximately twice the length of the shorter mesogen **2PhP**. The energy of each molecular structure was minimized by molecular modelling with the semiempirical method MOPAC/AM1 for the most extended conformers.

The preference for the SmA over the SmC phase, which was observed for all systems with a large length ratio, may also be explained by extensive out-of-layer fluctuations [2]. The molecules in a nontilted SmA phase can easily propagate into the next layer, which is favorable for structures of bidisperse molecules in which such out-of-layer fluctuations are essential to fill the free volume between the molecules of quite different size. In the tilted SmC phase, the core of the molecules is more tilted than the alkyl chains, which leads to a zig-zag shape of the molecules [3]. Due to this zig-zag shape, the molecules are “locked” in their layers and the free volume between them is no longer compensated. This costs a lot of free energy, which destabilizes the SmC phase in these bidisperse systems. For more detailed information see [2]. In our earlier studies [1–2] we were able to show the influence of strongly differing molecular lengths up to a length ratio of 1.8. The effect of an even larger length difference has not been studied yet, and it is unclear whether a larger length difference might lead to demixing. Therefore a system of two chemically similar phenylpyrimidines with a length ratio of 2 was investigated in this study, to clarify whether the effects of a length difference of 1.8 can be enhanced by a bigger length difference. We report herein that this system shows similar effects as the system with a length ratio of 1.8, but also the first signs of reduced miscibility. The long compound used in this study, **PhP16** (see below in Scheme 1 and Figure 2), exhibits a heretofore unidentified higher-ordered smectic phase [4], which we have identified by detailed investigations reported herein as a smectic F (SmF) phase.

## Results and Discussion

The liquid-crystalline materials used herein are shown in Scheme 1 and Figure 2. The component with long molecular length is the symmetric compound 2-(4-hexadecyloxyphenyl)-5-hexadecyloxypyrimidine (**PhP16**) [4]. It forms a broad SmC phase and a narrow higher-ordered smectic phase. Its molecular length was determined by molecular modelling as 50.6 Å. The component with short molecular length is the asymmetric compound 2-(4-butoxyphenyl)-5-octyloxypyrimidine (**2PhP**) [4]. It exhibits the typical liquid-crystalline phase sequence of nematic, smectic A and smectic C phases. Its molecular length is 25.6 Å, according to molecular modelling, which results in a **PhP16/2PhP** length ratio of almost 2.

**Figure 3:**
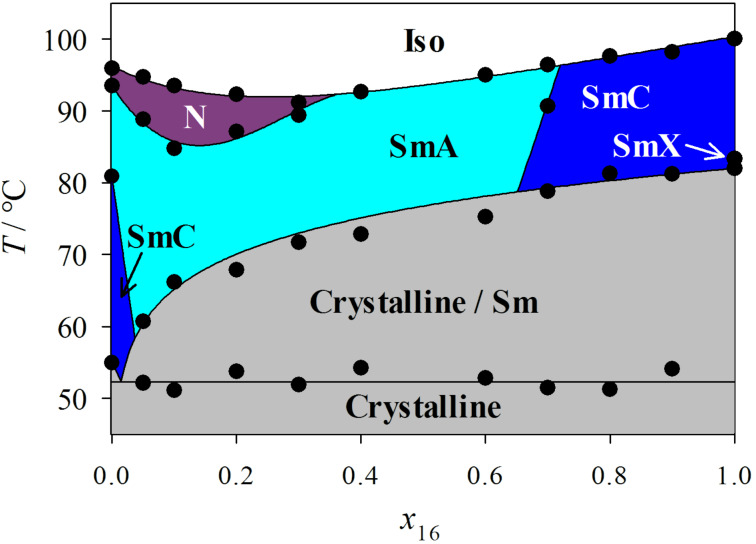
The phase diagram of the binary mixture system **PhP16**/**2PhP**.

First, the phase diagram of the system **PhP16**/**2PhP** was determined (Figure 3). This phase diagram is very similar to those reported in our earlier studies, with a length ratio of 1.8 [1–2]. Again, no destabilization of the smectic state was observed, and a eutectic point at approximately *x*_16_ = 0.015 was observed. However, the nematic phase disappears rapidly with increasing mole fraction *x*_16_ after a slight stabilization until *x*_16_ = 0.1, and it disappears at *x*_16_ = 0.4. In the smectic state, the SmA phase is much more stable than SmC. Although SmC is the dominant phase in both pure compounds, the SmC phase disappears after the addition of only 5 mol % of the long compound **PhP16**. On the other side of the phase diagram, the SmC phase is stable until a mole fraction of *x*_16_ = 0.6 is reached. Over a broad range of temperatures and concentrations, SmA is the dominant phase in the diagram.

**Figure 4:**
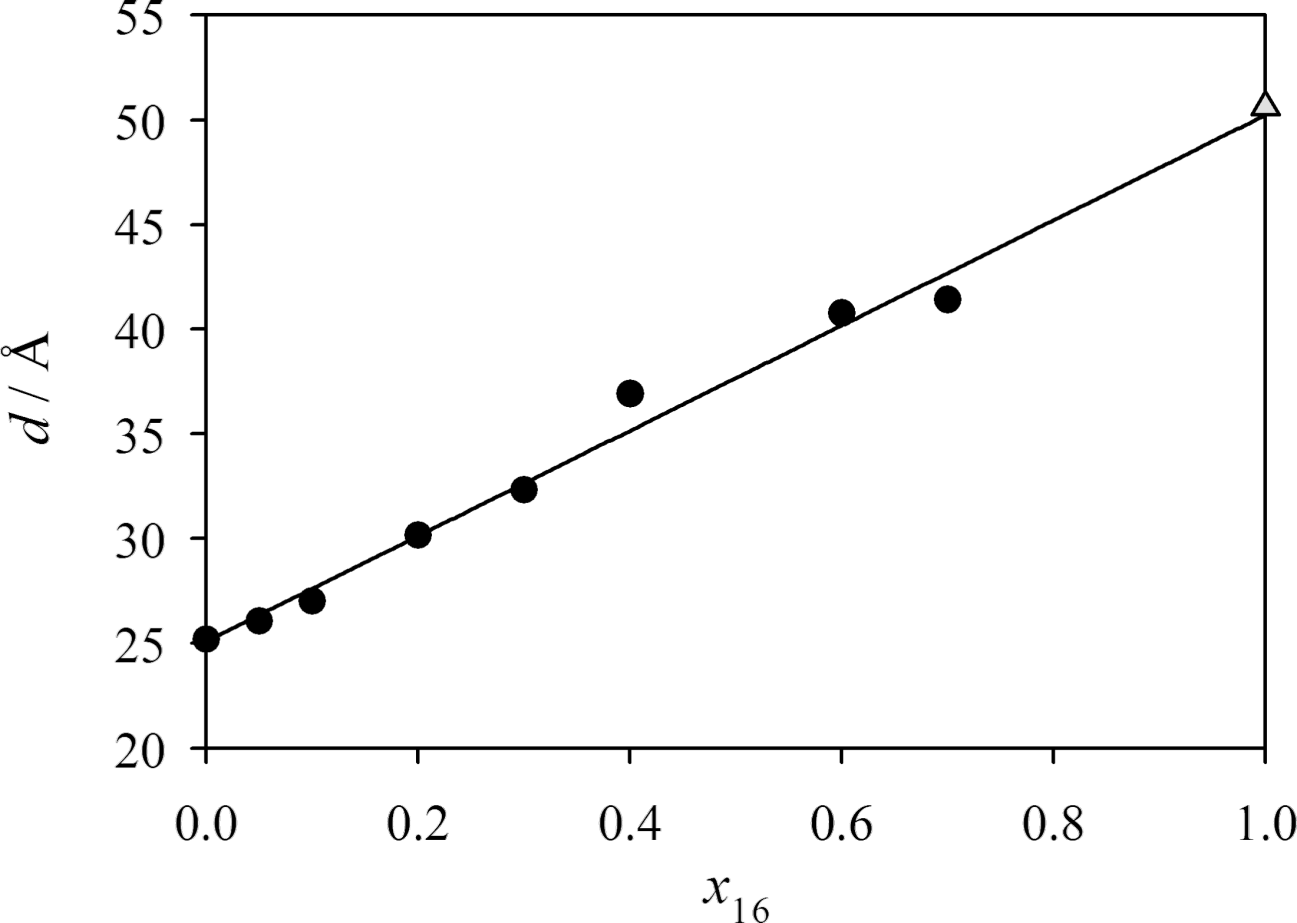
The layer spacing obtained by SAXS-measurements in the SmA phase at *T* = *T*_c_ is plotted against the mole fraction *x*_16_ (closed circles) as well as the molecular length for pure **PhP16** calculated by molecular modelling (grey triangle).

Small-angle X-ray scattering (SAXS) measurements were performed for all mixtures. As shown in Figure 4, the layer spacing *d* measured at *T* = *T*_c_ varies linearly with the mole fraction *x*_16_. This linear dependence suggests that the Diele additivity rule [5] can be applied for the mixtures of **2PhP** and **PhP16**. The Diele additivity rule states that the layer spacing of a mixture depends linearly on the layer spacings of the pure compounds and the mole fraction. According to Diele, the layer spacing *d* of a mixture of two pure compounds A and B can be calculated as *d* = *d*_A_·*x*_A_ + *d*_B_·*x*_B_. With this linearity of the smectic layer spacing, a value for the hypothetical SmA phase of pure **PhP16** is extrapolated to a value of *d* = 50.2 Å, which is in good agreement with the value of 50.6 Å calculated by using MOPAC/AM1 (Figure 2). For the short compound **2PhP**, there is also a good agreement between the calculated value of 25.6 Å for the extended length of the molecule and the experimentally determined *d*-value of 25.1 Å from SAXS.

**Figure 5:**
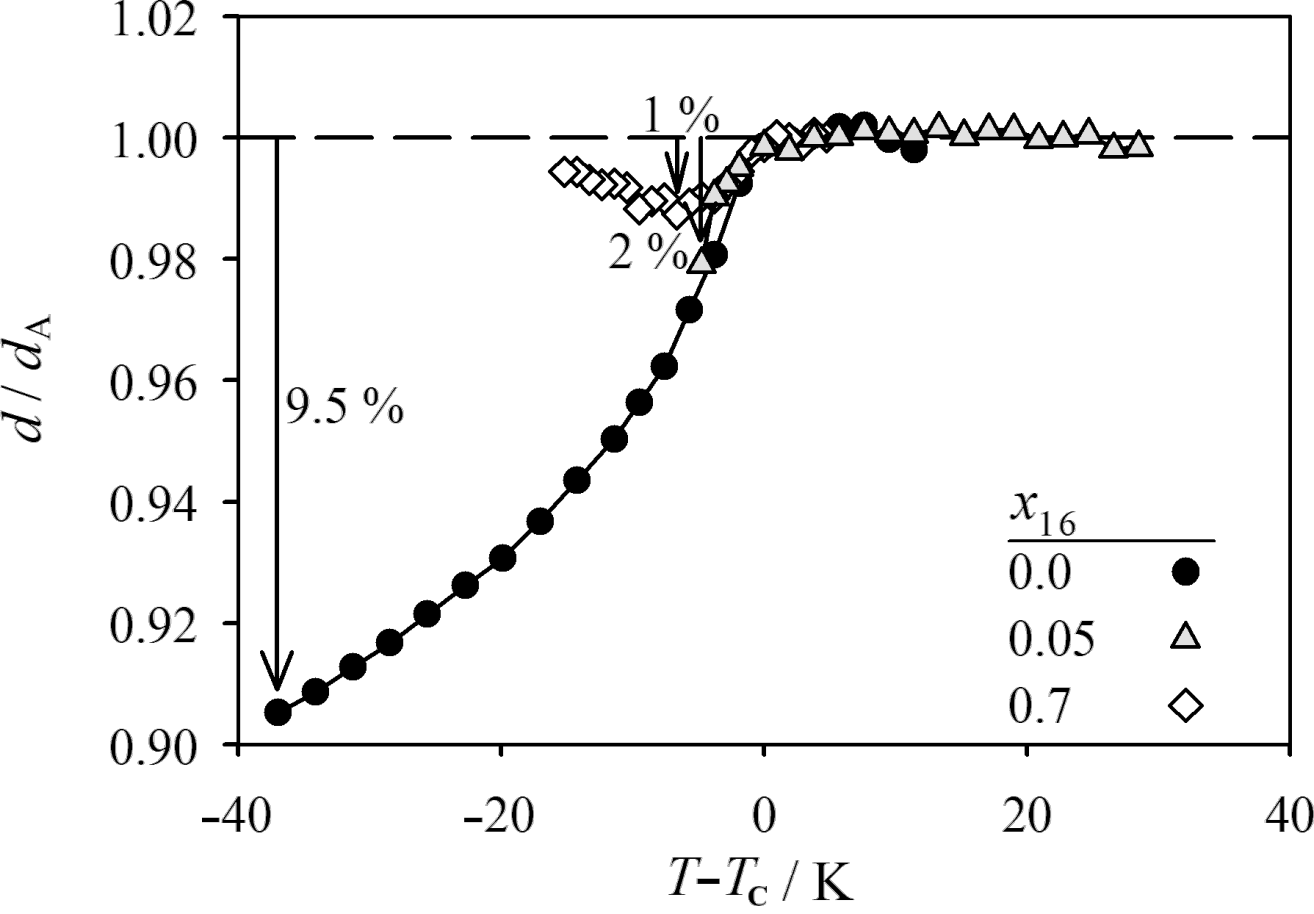
Reduced layer spacing *d*/*d*_A_ versus temperature difference to the phase-transition temperature *T*−*T*_c_ for pure **2PhP** (closed circles) and the two mixtures that show SmA and SmC phases with *x*_16_ = 0.05 (grey triangles) and 0.7 (open squares).

Figure 5 shows the reduced layer spacings *d*/*d*_A_ (where *d*_A_ is the layer spacing of a hypothetical SmA phase in the temperature range of the SmC phase, calculated by extrapolation of the temperature-dependent layer spacing of the SmA phase) versus *T*−*T*_c_ of the pure compound **2PhP** as well as for the mixtures with *x*_16_ = 0.05 and 0.7. Compound **2PhP** shows a substantial maximum layer shrinkage of 9.5%, whereas the mixture with *x*_16_ = 0.05 shows a narrow SmC phase and a maximum layer shrinkage of only 2% at *T*−*T*_c_ = −5 K, which is similar to the layer shrinkage of **2PhP** at the same reduced temperature. The mixture with *x*_16_ = 0.7 shows only a small maximum layer shrinkage of 1% at *T*−*T*_c_ = −8 K.

**Figure 6:**
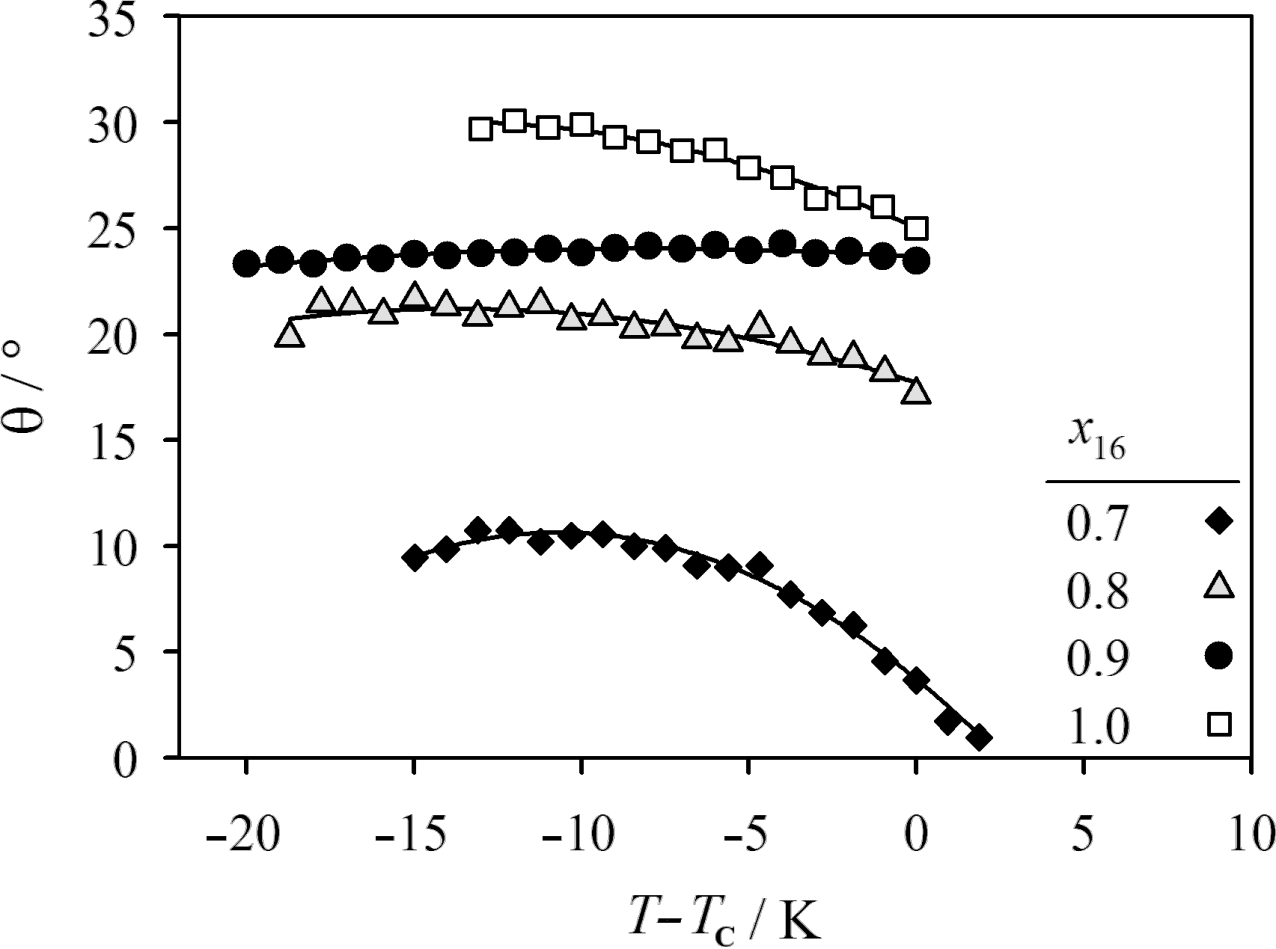
The tilt angle θ is plotted against the temperature difference to the phase-transition temperature *T*−*T*_c_ for the mixtures with *x*_16_ = 0.7 (filled diamonds), 0.8 (grey triangles), 0.9 (filled circles) and the pure compound **PhP16** (open squares).

Figure 6 shows the optical tilt angles of the mixtures, which were determined in the ferroelectric SmC* phase [6–7] to gain a better understanding of the unusual behavior of the layer spacing. To obtain a chiral SmC* phase, 4 mol % of the chiral dopant (*R,R*)-2-(4-octylphenyl)-5-(2,3-difluorohexyloxy)pyridine (**MDW510**, Scheme 1) [8–9] was added. Optical tilt angles were measured for the mixtures with *x*_16_ = 1.0, 0.9, 0.8 and 0.7; the largest tilt angles were observed for the pure compound **PhP16**. The progressive addition of the short compound caused a reduction of the tilt angle from a value of ca. 25° for *x*_16_ = 0.9 to ca. 10° for *x*_16_ = 0.7. Further addition of the short compound caused the tilt angle to vanish completely, which corresponds to a concentration-induced phase transition from the SmC to the SmA phase. Optical tilt angles are compared with the tilt angles calculated from the X-ray layer shrinkage by using the expression θ = cos^−1^(*d*_C_/*d*_A_), and are in good agreement. This shows that the small layer shrinkage observed in the mixtures correlates to the small tilt angles, and that the mixtures do not exhibit so-called “de Vries-type” behavior [10].

**Figure 7:**
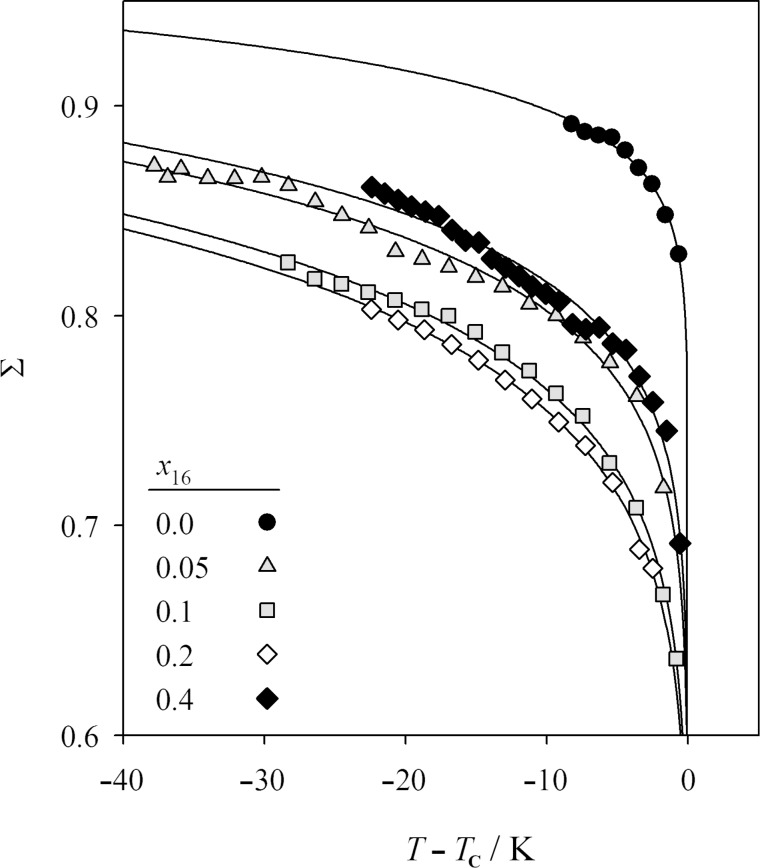
Translational order parameter Σ in the SmA phase versus temperature difference to the phase-transition temperature *T*−*T*_c_ for the mixtures with *x*_16_ = 0.4 (filled diamonds), 0.2 (open diamonds), 0.1 (grey squares) and 0.05 (grey triangles) and for the pure compound **2PhP** (filled circles).

The translational order parameter Σ [11–12] was measured in the SmA phase to give a measure of the degree of molecular ordering in the layers. Σ is defined as the amplitude of the density wave that originates from the 1-D periodic smectic layers [13]. In Figure 7, the smectic order parameters Σ for the pure compound **2PhP** and the mixtures with *x*_16_ = 0.05, 0.1, 0.2 and 0.4 are shown. The highest smectic order parameters were found for the pure compound **2PhP** in the range of 0.9. The addition of a small amount of **PhP16** reduced the smectic order to values around 0.85. Adding more **PhP16** caused further reduction in Σ to values around 0.8 for the *x*_16_ = 0.1 and 0.2 mixtures. Smectic order was partially recovered upon adding more **PhP16** to a mole fraction *x*_16_ = 0.4. The smectic order thus considerably reduced upon the loss of the SmC phase in the phase diagram, and was partially recovered upon re-entry of the SmC phase into the phase diagram.

Besides a SmC phase, the pure material **PhP16** also forms a heretofore unidentified higher-ordered smectic phase [4]. After performing a detailed investigation of this material, we determined that this higher-ordered smectic phase in **PhP16** is a smectic F (SmF) phase, which is a tilted version of the hexatic smectic B phase. In the SmF phase, the tilt direction of the molecules is towards a face of the quasihexagonal net, i.e., halfway between nearest neighbors [14].

**Figure 8:**
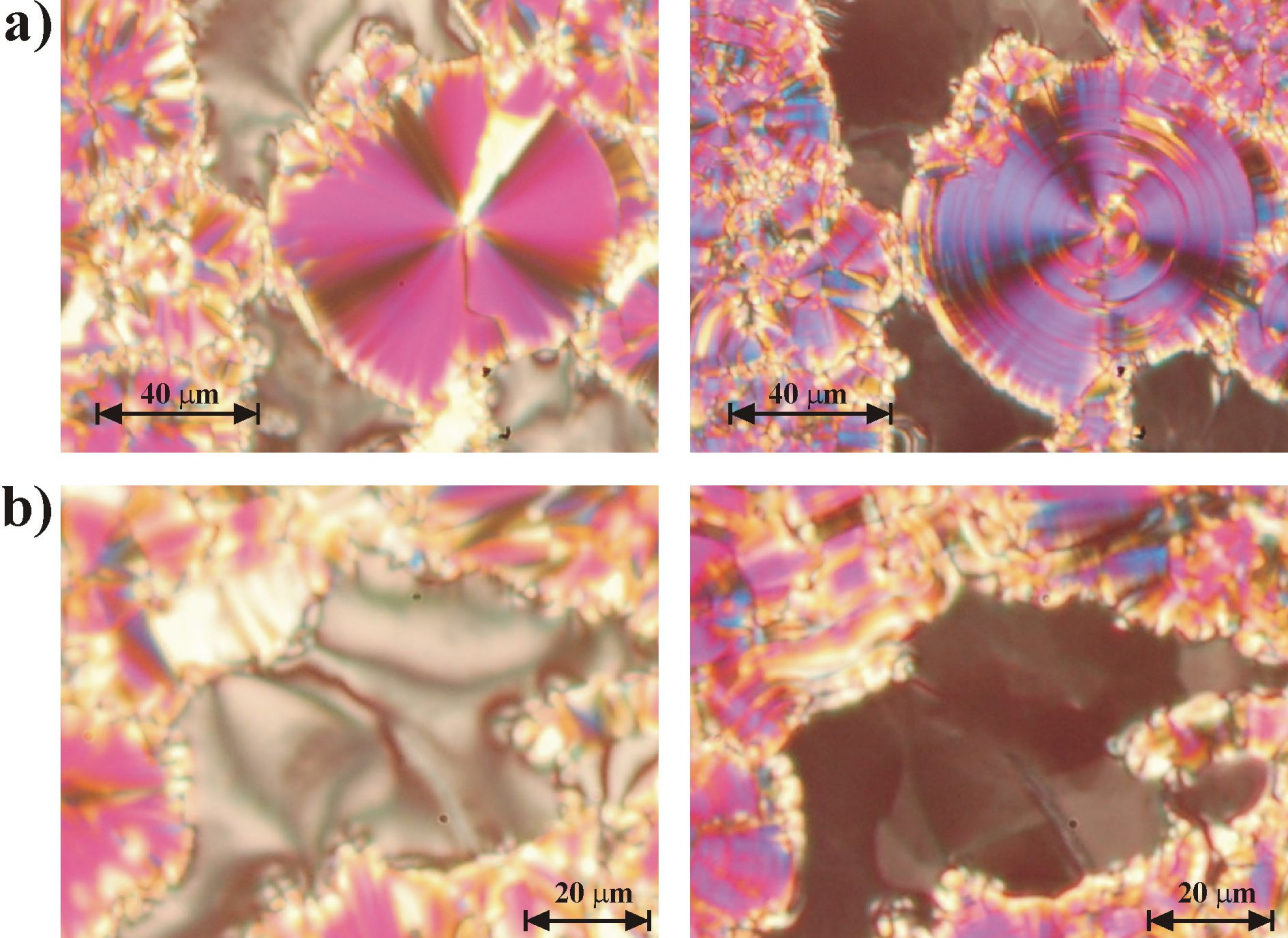
Textures of the pure compound **PhP16** on cooling. Left: SmC phase at 96.8 °C Right: same positions in the SmF phase at 82.7 °C. (a) In the upper part a fan-shaped texture is shown, while (b) in the lower part a homeotropic texture can be seen.

The textures of the higher-ordered SmF phase were investigated by polarizing microscopy. Micrographs of the textures of the SmC and SmF phase of **PhP16** are shown in Figure 8 for samples with partial planar (Figure 8a) and partial homeotropic alignments (Figure 8b). The planar aligned SmC phase shows a fan-shaped texture; after cooling of the sample to the SmF phase, a slight change of colors was observed, which indicates a slight increase in birefringence, and the fans show circular stripes. The homeotropic regions shown in Figure 8b exhibit a Schlieren texture characteristic of the SmC phase. In the SmF phase, a mosaic texture is observed. This homeotropic mosaic texture is a clear indication that the higher-ordered smectic phase is a tilted phase, since the homeotropic regions would be completely black for a nontilted phase. This homeotropic mosaic texture together with the planar circularly striped fan texture is typically found for the soft-crystalline G phases as well as for SmF phases [15]. The texture observations suggest that the higher-ordered smectic phase must be a tilted phase. A demonstration that the sample is shearable ruled out soft-crystalline phases (E, G, H, J and K), leaving only the two fluid, higher-ordered, hexatic smectic phases SmF and SmI as possibilities.

**Figure 9:**
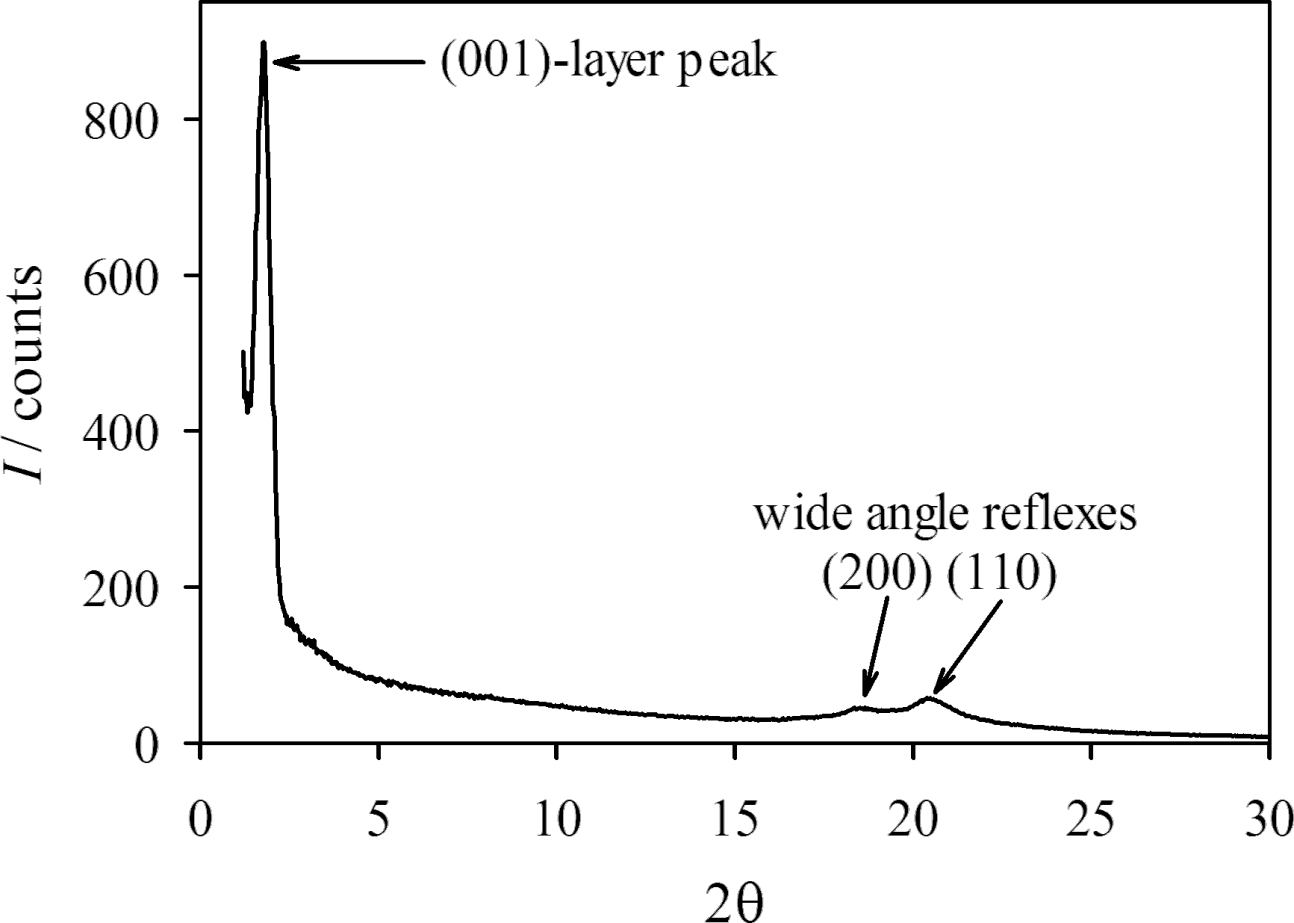
Integrated intensity profile of an unoriented sample of **PhP16** at *T* = 82.5 °C in the SmF phase.

To distinguish between these two hexatic smectic phases, wide-angle X-ray scattering (WAXS) was performed in the higher-ordered smectic phase of **PhP16**. We were able to investigate the sample in three different configurations: (i) as an unoriented sample, (ii) with the smectic layer normal *k* oriented parallel to the incident X-ray beam, and (iii) with the smectic layer normal mainly oriented perpendicular to the X-ray beam. Figure 9 shows the integrated intensity profile of an unoriented sample of the SmF phase of **PhP16** at 82.5 °C, which shows three peaks. The (001)-layer peak occurs at a scattering angle 2θ = 1.77°, which gives a layer spacing *d* of 49.6 Å. This value differs from the value obtained from the SAXS measurements (see below in Figure 12a), which is due to the fact that the WAXS measurements are not very precise in the SAXS regime. Two distinct wide-angle peaks are observed at 2θ = 18.4 and 20.5°, which clearly indicate that the unidentified phase is a fluid hexatic phase, as the less-ordered smectic phases such as SmA and SmC would show a diffuse halo, and the more ordered 2-D soft-crystalline phases such as G and E would show more peaks. In the case of the SmF phase, the two peaks observed are the (200) and the (110) peaks. As the SmF phase is a tilted hexatic phase, it can be described by a centred monoclinic unit cell, with the three axes *a*, *b* and *c* and the angle β ≠ 90° [14]. The *c*-axis, which describes the height of the unit cell, is tilted at the angle β = (90° + tilt angle), with respect to the plane spanned by the *a*- and *b*-axes [14]. The unit cell parameters of the 2-D hexatic lattice can now be calculated from the (001), (200) and (110) peaks. The received parameters are *a* = 10.84 Å, *b*= 4.86 Å, *c* = 56.21 Å and β = 128°.

**Figure 10:**
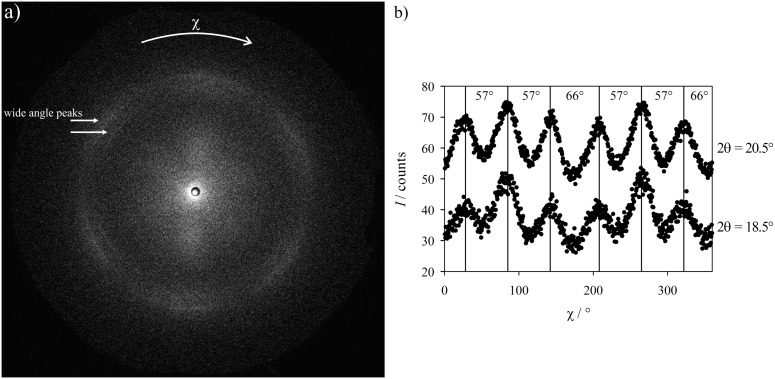
(a) WAXS-measurement of the SmF phase of **PhP16** at *T* = 82 °C with the smectic-layer normal **k** oriented parallel to the incident beam. Two wide-angle peaks can be observed, but no layer peak. (b) Integration of the two WAXS peaks in dependence of the azimuthal angle χ.

WAXS measurements on a sample with the smectic-layer normal **k** oriented parallel to the X-ray beam were then performed. Figure 10 shows the 2-D diffractogram of the oriented SmF phase at *T* = 82 °C as well as the azimuthal distribution of the integrated intensity of the two wide-angle peaks. In the 2-D diffractogram (Figure 10a), no layer peak could be observed as the layer normal was oriented parallel to the beam. Again the two wide-angle peaks, which could also be seen in the unoriented sample at 2θ = 18.5 and 20.5°, are observed. They show a pseudohexagonal intensity distribution on dependence of the azimuthal angle χ. The behavior of their intensity versus the angle χ can be seen in Figure 10b. The two wide-angle peaks show six maxima separated from each other by two different angles (57 and 66°), which reflects the pseudohexagonal structure of a SmF or SmI phase.

**Figure 11:**
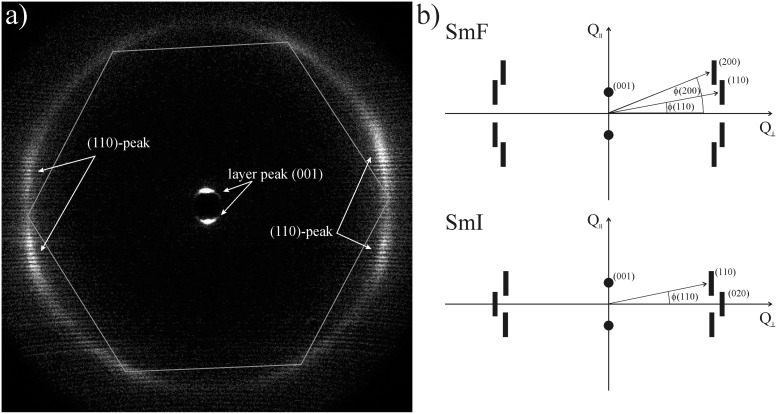
(a) WAXS-measurement of the SmF phase of **PhP16** at *T* = 88 °C with the smectic layer normal oriented mostly perpendicular to the incident beam. Only one wide-angle peak can be observed as well as the (001) layer peak. (b) Diffraction patterns of the SmF and SmI phase with the incident beam perpendicular to the layer normals (adapted from [14]).

**Figure 12:**
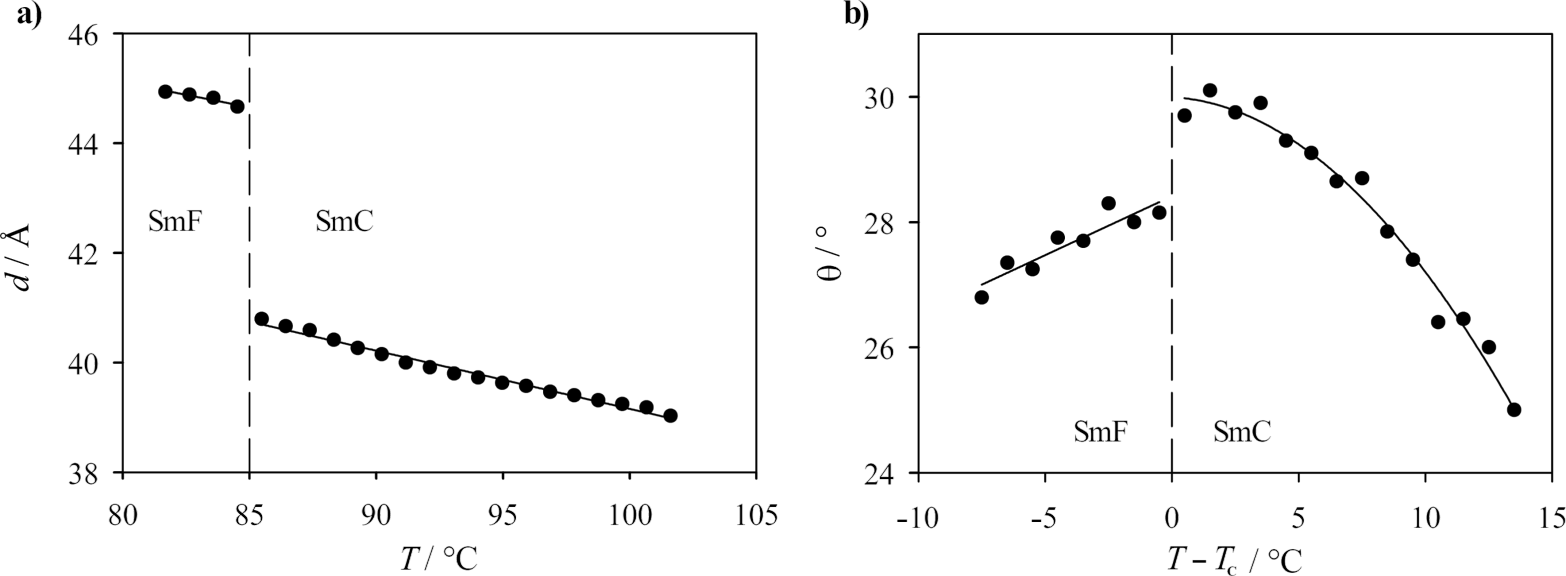
(a) Layer spacing of the SmF and the SmC phase of the pure compound **PhP16** in dependence of the temperature *T*. (b) Tilt angle θ versus reduced temperature *T*−*T*_c_ for the pure compound **PhP16** in the SmF and in the SmC phase.

In the 2-D diffractogram of the SmF phase of **PhP16** shown in Figure 11a, the smectic layer normal is mostly oriented perpendicular to the incident X-ray beam. In this geometry the (001) layer peak at 2θ = 1.82° as well as the (110) peak at 2θ = 20.4° were observed. We did not observe the second (200) peak, which was observed in Figure 9 at 2θ = 18.5°. This could be due to the fact that the (200) peak was less intense than the (110) peak at 2θ = 20.5° and that a different X-ray setup was used for the measurement in Figure 11a. Therefore, it may be due to the other setup that we could not detect the second, less-intense wide-angle peak. The sample used for the measurement in Figure 11a was very well oriented, as can be seen from the two sharp (001) layer peaks in the small-angle regime. The diffraction pattern in Figure 11a allows us to distinguish between SmF and SmI. Their diffraction patterns are presented schematically in Figure 11b. In the case of a SmF phase, the radial integration of the outer peak (the 110 peak) gives four maxima at the angles χ = sin^−1^(±0.5 sin θ) to the equator, with θ being the tilt angle, whereas the integration in dependence of χ of the outer peak (the 020 peak) of a SmI phase would result in only two maxima, which are located at the equator [14]. In the diffraction pattern of the higher-ordered smectic phase of **PhP16** in Figure 11a, four intense maxima are found at the angles χ = 75, 107.5, 257 and 286°. These four maxima, which are not located at the equator, clearly indicate that the higher-ordered smectic phase of **PhP16** is a SmF phase. The average angular distance of the four maxima to the equator, which is defined by the position of the maxima of the small angle peaks, is χ =15.4°. The tilt angle derived from this measurement is θ = 32°, which corresponds quite well with the optical tilt angle of 28.2° in the SmF phase (see Figure 12b). There are four other less-intense maxima observed on the radial intensity distribution of the (110) wide-angle peak: χ = 31, 154, 209 and 333°. This shows that the layer normal **k** is not oriented perpendicular to the incident X-ray beam throughout the whole sample, but in some parts it is also oriented parallel. This parallel orientation gives a pseudohexagonal intensity distribution, as seen in Figure 10a. As the intensity of these peaks is much smaller than the one of the peaks coming from the perpendicular orientation, only four of the six corners of the pseudohexagon can be seen, as the other two are underneath the other peaks. In Figure 11a the pseudohexagon is marked with white lines.

Precise measurements of the smectic layer spacing *d* were obtained by small-angle X-ray scattering (SAXS). Figure 12a shows the smectic layer spacing *d* of the SmC and the SmF phases of **PhP16**. At the phase transition, we observe a clear step of the layer spacing from about 40 Å in the SmC phase to higher values of about 45 Å in the SmF phase, which suggests that the tilt angle is reduced at the phase transition. Accordingly, the optical tilt angle θ of **PhP16** was measured (Figure 12b); the tilt angle in the SmF phase is ca. 27° whereas it reaches 30° in the SmC phase. From the layer spacing *d*_F_ in the SmF phase and its tilt angle θ, the molecular length *L* of **PhP16** is calculated as *L* = *d*_F_/cos θ. With *d*_F_ = 44.7 Å and θ = 28.2°, the effective molecular length *L* is 50.7 Å, which is in good agreement with the molecular length of 50.6 Å obtained by molecular modelling. In conclusion, the material **PhP16** shows a first-order phase transition from the tilted SmC phase to the tilted SmF phase at the transition temperature *T*_c_.

## Conclusion

The investigations of the phase diagram of the two phenylpyrimidines **PhP16** and **2PhP** with a length ratio of 2 is consistent with our previous study of a similar binary system with a length ratio of 1.8 [1–2]. The phenylpyrimidine **PhP16** used in this study has a greater length than the compound **PhP14** that has been used in our earlier studies. The greater length of **PhP16** results in a different phase sequence, as **PhP16** forms a higher-ordered smectic phase in addition to the SmC phase, which is also formed by **PhP14**. Detailed investigations of this higher-ordered phase by texture analysis, shearing, and tilt-angle measurements as well as small- and wide-angle X-ray scattering confirmed that it is a smectic F phase. The greater length of **PhP16**, relative to **PhP14**, also changes the behavior of the mixtures with **2PhP**. In the system **PhP16**/**2PhP**, with the greater length difference, the stabilization of the smectic state is less pronounced. This indicates that the stability of the smectic state decreases with increasing length difference between the two molecules. This suggests that there is an optimum length difference, which is around 1.8, maximizing the stability of the smectic state.

## Experimental

The compound **PhP16** [4] as well as the compound **MDW 510** [8] were synthesized according to the published procedures and showed the expected physical and spectral properties. The liquid-crystalline compound **2PhP** was purchased from a commercial source. A Kratky compact camera from Anton Paar was used to perform small-angle X-ray scattering (Ni-filtered Cu K_α_ radiation with wavelength λ = 1.5418 Å). The unaligned samples were filled into Mark capillary tubes of 0.7 mm diameter and put into a temperature-controlled sample holder (Anton Paar). A one-dimensional electronic detector by M. Braun was used to record the scattered intensity. Polarizing microscopy was performed by using a Leica DM-LP polarizing microscope with an Instec HS1-i hot stage. For the determination of optical tilt angles, the achiral samples were doped with 4 mol % of the chiral compound **MDW510** and filled into glass cells with a spacing of 1.6 μm, which were coated with a rubbed Nylon/ITO surface (AWAT PPW, Poland). The tilt angle θ was measured at a field strength of 12.5 V·μm^−1^.
